# Automated identification of chalcogen bonds in AlphaFold protein structure database files: is it possible?

**DOI:** 10.3389/fmolb.2023.1155629

**Published:** 2023-07-06

**Authors:** Oliviero Carugo, Kristina Djinović-Carugo

**Affiliations:** ^1^ Department of Chemistry, University of Pavia, Pavia, Italy; ^2^ Max Perutz Labs, Department of Structural and Computational Biology, University of Vienna, Vienna, Austria; ^3^ European Molecular Biology Laboratory (EMBL) Grenoble, Grenoble, France; ^4^ Department of Biochemistry, Faculty of Chemistry and Chemical Technology, University of Ljubljana, Ljubljana, Slovenia

**Keywords:** AlphaFold, chalcogen bond, 3D structure prediction, stereochemical criteria, experimental 3D structure

## Abstract

Protein structure prediction and structural biology have entered a new era with an artificial intelligence-based approach encoded in the AlphaFold2 and the analogous RoseTTAfold methods. More than 200 million structures have been predicted by AlphaFold2 from their primary sequences and the models as well as the approach itself have naturally been examined from different points of view by experimentalists and bioinformaticians. Here, we assessed the degree to which these computational models can provide information on subtle structural details with potential implications for diverse applications in protein engineering and chemical biology and focused the attention on chalcogen bonds formed by disulphide bridges. We found that only 43% of the chalcogen bonds observed in the experimental structures are present in the computational models, suggesting that the accuracy of the computational models is, in the majority of the cases, insufficient to allow the detection of chalcogen bonds, according to the usual stereochemical criteria. High-resolution experimentally derived structures are therefore still necessary when the structure must be investigated in depth based on fine structural aspects.

## Introduction

In 2021, sensational progress was made in protein structure prediction with AlphaFold2, the artificial intelligence system developed by DeepMind (Jumper et al., 2021). These predictions became freely available in the AlphaFold Protein Structure Database (AlphaFold DB), created by EMBL-EBI, which presently includes more than 200 million predictions (Tunyasuvunakool et al., 2021) (Varadi et al., 2022).

The reliability of these predictions is astonishing, and–even more critical–the reliability is estimated at the level of each single amino acid through the predicted local distance difference test (pLDDT), enabling users to identify structures’ moieties that might be uncertain, e.g., conformational disorder. The importance of this precise accuracy estimate has been documented recently in a survey of AlphaFold2 applications in structural and molecular biology (Akdel et al., 2022).

Understandably, these predictions have been scrutinized from different perspectives. Some may find them unsatisfactory because based on statistics and not on physics and chemistry first principles (Moore et al., 2022), others suggest that they cannot, at least for the moment, reach the accuracy of experimental structures (Shao et al., 2022). Others have observed that they are insufficient in dealing with structural disorder or aggregation (Pinhero et al., 2021) and with structural features independent of gene sequence, like, for example, structuration of disordered proteins upon ligand binding or protein solvation by membrane lipids (Azzaz and Fantini, 2022). It has also been observed that other prediction methodologies, particularly homology modeling, can give equally good results in many cases at a much lower computational cost (Lee et al., 2022). Akdel and colleagues observed that the impact of missense mutations on the thermodynamic stability of proteins can be predicted equally well by using experimental structures or AlphaFold2 models (Akdel et al., 2022), though AlphaFold2 does not seem to be appropriate to forecast the structure of mutated proteins (Buel and Walters, 2022) (Pak et al., 2021). Naturally, the next desired level of prediction is on protein-protein, protein-nucleic acid and protein-ligand interactions. Fine progress on prediction of protein-protein interactions and automatization of pipelines has been made with AlphaFold-Multimer and AlphaPullDown (Evans et al., 2022) (Bryant et al., 2022) (Humphreys et al., 2021) (Mosalaganti et al., 2022) (Yu et al., 2023), for protein-protein interactions and as well as on generation of models loaded with their ligands (Hekkelman et al., 2022). Recently, AlphaFold2 models have also been used for validation of experimental models (Sanchez Rodriguez et al., 2022), computational docking (Holcomb et al., 2023), and cryo-EM refinements (Terashi et al., 2023).

Here, we seek to assess the degree to which these computational models can provide information on subtle details that may be important in various applications in protein engineering, chemical biology, and biotechnology. As an example, we focus on chalcogen bonds (referred to as ChB according to (Aekeroy et al., 2019)) formed by disulphide bridges (Aekeroy et al., 2019) (Vogel et al., 2019). This is an interesting test case because chalcogen bonds are not yet parameterized in any molecular mechanics/dynamics force field, and consequently, their presence cannot be affected by energy minimization protocols. In other words, these moderate clashes can be tolerated if compensated by a good and native-like packing around them, involving attractive interactions like hydrogen bonds, van der Waals interactions etc.

A ChB is an attractive interaction similar to a hydrogen bond, where a nucleophilic atom is attracted by an element of group 16 heavier than oxygen (chalcogens; sulphur, selenium and tellurium). While in proteins the most abundant chalcogen atom is sulphur—selenium is very rare and tellurium absent—there are several nucleophiles—oxygen, sulphur, and aromatic rings (Aekeroy et al., 2019; Scilabra et al., 2019; Carugo et al., 2021).

**FIGURE 1:**
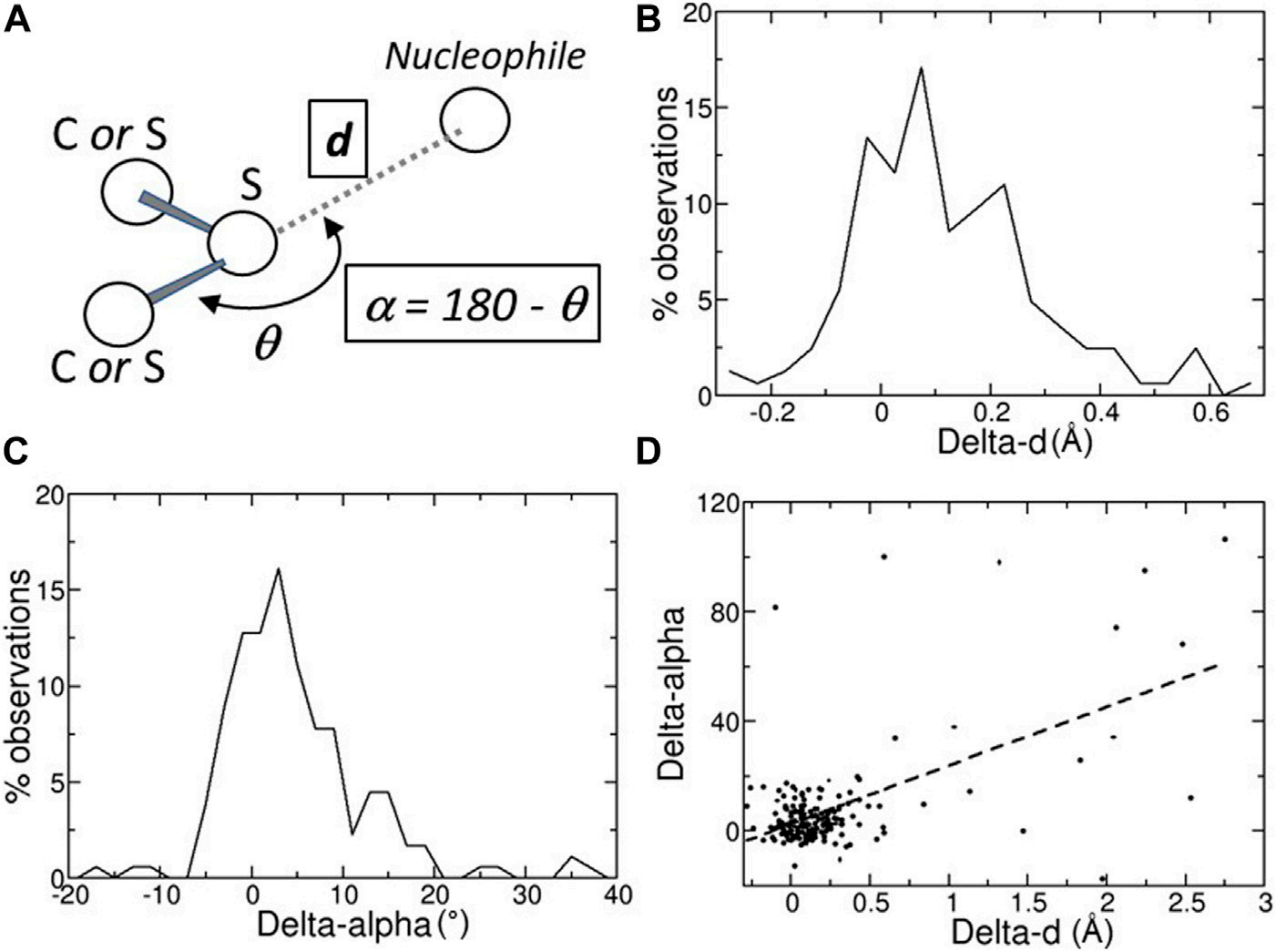
**(A)** Schematic representation of a chalcogen bond; the position of the nucleophilic atom, for example, a main-chain oxygen atom, relative to the sulfur atom is monitored with two variables, *d*, its distance from the sulfur atom, and *α* = 180 – *θ*, where *θ* is the angle defined by the nucleophile, the sulfur, and the atom covalently bound to the sulfur. **(B)** Distribution of the Delta-*d*s, the differences between the distances *d* observed in the predicted structures (Alpha Fold DB) and in the experimental structures (PDB). **(C)** Distribution of the Delta-*α*s, the differences between the angles *α* observed in the predicted structures (Alpha Fold DB) and in the experimental structures (PDB). **(D)** Scatter plot of the Delta-*α versus* the Delta-*d* values. Delta-*d* and Delta-*α* values are given in Å and degrees, respectively.

In the interacting moiety (C,S)-S…Nu (Figure 1A), where Nu is a nucleophile, an electrostatic attraction between the positively charged region found along the prolongation of the C-S bond can stabilize the interaction (Pascoe et al., 2017); additionally a n→σ* orbital delocalization between the nucleophile lone pair and the anti-bonding orbital of C-S may occur (Pascoe et al., 2017). The strongest ChBs may compare to conventional H-bonds (Pascoe et al., 2017), though it has been shown that in proteins hydrogen bonds tend to prevail over ChBs (Carugo, 2023).

ChBs are an interesting test case because they are not yet parameterized in any molecular mechanics/dynamics force field, and consequently, their presence cannot be favoured by energy minimization protocols. On the contrary, they would be considered inter-atomic clashes and consequently disfavoured. In other words, these moderate clashes can be tolerated if compensated by a good and native-like packing around them, involving attractive interactions like hydrogen bonds, van der Waals interactions etc.

## Materials and methods

### Data selection

All experimental structural data were taken from the Protein Data Bank (PDB) (Bernstein et al., 1977) (Berman et al., 2000) (wwPDB Consortium, 2019). Only X-ray crystal structures refined at a resolution better than (or equal to) 1.5 Å and determined in the 90–110 K temperature range were retained. Care was taken to discard multi-model refinements and structures where more than 5% of the atoms are not protein or water atoms. Then, pairwise percentage of sequence identity was limited to 40% with CD-HIT (Li and Godzik, 2006) (Fu et al., 2012), to avoid redundancy.

Computational models were taken from the AlphaFold Protein Structure Database (Tunyasuvunakool et al., 2021) (Varadi et al., 2022). They were identified through the sequence database accession codes, reported in the DBREF of the PDB files, which allow to identify the computational model with exactly the same sequence of the experimental structure. In this way, we ensured to compare molecules that share the same chemical formula. Not all PDB files have a DBREF annotation, typically mutants which are not in UniProt (Wu et al., 2006) and in AlphaFold DB (Tunyasuvunakool et al., 2021) (Varadi et al., 2022).

### ChBs detection

A ChB can be formed by a nucleophilic atom and a chalcogen atom that is covalently bound to another atom (Figure 1A). The nucleophilic atom must be positioned along the prolongation of the covalent bond, or along the prolongation of one of the two covalent bonds if the chalcogen is divalent. As a consequence, there are two parameters that must be monitored, the distance *d* between the nucleophilic and the chalcogen atom and the angle *α* (Figure 1A).

According to the IUPAC recommendations, the distance d must be shorter than the sum S of the van der Waals radii of the nucleophilic and chalcogen atoms (Aekeroy et al., 2019), despite the fact that the use of van der Waals radii in determining non-bonding interactions may need to be revised (Politzer et al., 2007). Here, to account for the lower accuracy of macromolecular structures relative to small molecule structures, we added a small margin to S and, consequently, a in a ChB d must not be larger than S + 0.1 Å.

The angle *α*, which is supplementary to the angle *θ*, must be as small as possible. In chemical crystallography and material science, a ChB is usually characterized by a value smaller than 20°. Here, to account for the lower accuracy of macromolecular structures, we increased this threshold value to 25°.

Similar settings were previously used (Carugo, 2023) (Carugo, 2023) and can be compared with estimated average positional standard errors of 0.046 Å, which imply estimate errors of about 0.06 Å on bond distances and of about 2° on bond angles.

## Results and discussion

About one-half (43%) of the ChBs observed in the experimental structures are present in the computational models if the same stereochemical criteria are used. This means that about one-half of the ChBs observed experimentally in high resolution crystal structures are not observed in the models deposited in AlphaFold DB.

Does this indicate that these models are wrong? Not really. On the contrary, models available in AlphaFold DB are extremely similar to the experimental crystal structures.

In many cases, the chalcogen-nucleophile contacts cannot be recognized as ChB because they are slightly longer than the experimental ones. In fact, the predicted distances *d* and angles *α* are close to those observed experimentally, with the differences between predicted and experimental *d* (Delta-*d*) and *α* (Delta-*α*) are not very far from 0 (Å or °; Figures 1B,C ). The average absolute values of Delta-*d* and Delta-*α* are equal to 0.28 ± 0.04 Å and 9.5° ± 1.3°, respectively. These values must be compared to the estimated positional standard errors (Dinesh Kumar et al., 2015), which are very small (0.046 ± 0.002 Å) as expected for high-resolution structures.

Furthermore, the average absolute value of Delta-d is about three times greater than that calculated on the contacts (shorter than 3.5 Å) between main-chain oxygen and nitrogen atoms involving the residues that form ChBs (0.106 ± 0.008 Å). This clearly reveals that, within the same structural region, ChB predictions are much less accurate than main-chain hydrogen bond predictions.

**FIGURE 2:**
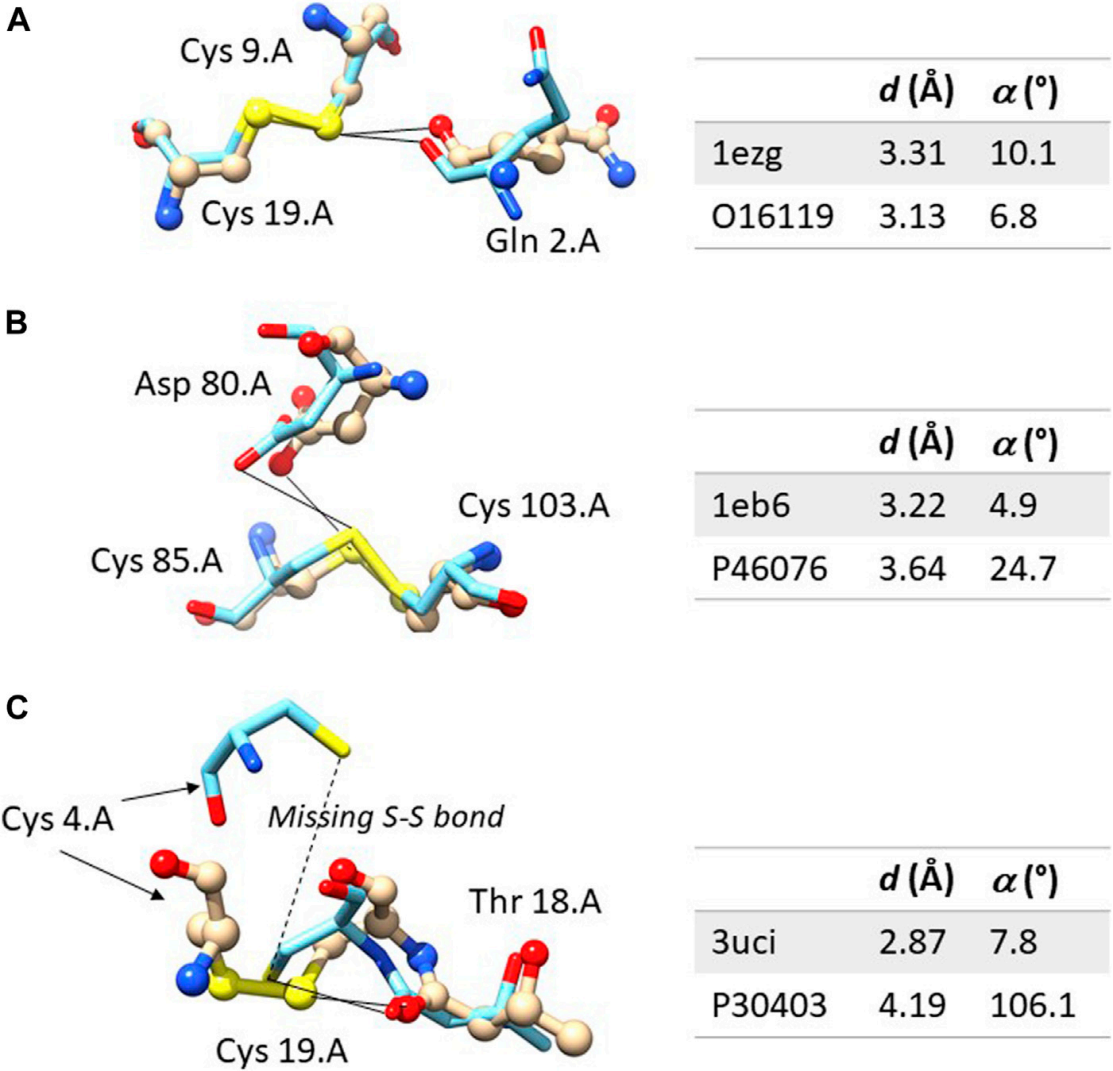
Comparison between the experimental and computation ChBs in three selected examples. The experimental structure is represented with ball and sticks and the computational model only with sticks. In **(A)** the ChB is detectable in both structures; in **(B)**, the ChB is detectable only in the experimental structures since the distance *d* is slightly too long in the computational model; in **(C)**, the ChB is detected in the experimental structures and it cannot be detected in the computational model where the disulphide bond is absent–this is a region partially unstructured of the protein. Color code: nitrogen is blue, oxygen red, sulfur yellow, carbon light grey in the experimental structure and azure in the AlphaFold DB model. For each residue, the following information is given: name, sequence number and chain identifier. The figure was prepared with Chimera 1.16.

Figure 2A shows an example in which the ChB is detected in both the experimental structures and in the computational model. The sulfur-oxygen distance *d* is even shorter in the AlphaFold DB model and the angle *α* is even closer to 0° in the AlphaFold model. Figure 2B shown an example in which the ChB is detected in the experimental structure and not in the AlphaFold DB model. The local stereochemistry is quite well predicted but the sulfur-oxygen distance is slightly too long (the threshold is sum of the van der Waals radii, 1.52 Å for oxygen and 1.80 Å for sulfur, with and small positive tolerance of 0.1 Å, is 3.42 Å). This might seem a minor distortion, but in terms of chemical interactions is crucial.

In principle, it would be possible to increase the threshold values of d and α that allow to automatically detect ChBs in such a way to increase the number of ChBs in the models of AlphaFold DB. However, the values of these thresholds strictly depend on the laws of chemistry and physics and nothing indicates here that this is justified. AlphaFold2 is a powerful tool for predicting protein three-dimensional structures.

There are only a few cases where the predicted structure is very different from the experimental one. For example, in ten cases with Delta-*α* > 30°, most of them have large Delta-*d*s (Figure 1D). However, in seven of them (PDB: 1vf8, 3b4n, 3om0, 3uci, 6ac5, 6h20, and 6jk4), the average pLDDT values of the residues bridged by the ChB are <90, indicating that side-chains might not be predicted reliably, or even <50, suggesting that predictions should be treated with caution. Only three predicted structures (PDB: 3soj, 4kl3, and 6ya1) have pLDDT >90, suggesting that models have high accuracy, despite Delta-*α* >30°.

Figure 2C shows one of the rare examples where AlphaFold DB models seem to be completely inadequate. The local stereochemistry–this a partially disordered part of the protein–is wrong, the cysteine 4 is misplaced and the disulphide bonds is broken. No surprisingly, the ChB is broken, too.

We conclude that computational models produced with AlphaFold2 and stored in AlphaFold DB are accurate–we note that for these proteins a high-resolution crystal structure is available in the PDB. In the majority of the cases, they show contacts between sulphur atoms of disulphide bridges and protein nucleophilic atoms that are comparable to the experimental ones. However, the accuracy of the computational models is, in the several cases, insufficient to allow the detection of ChBs, according to the usual stereochemical criteria.

This indicates that high-resolution structures are still necessary when the structure must be investigated in depth and when the importance of weak interactions, like ChBs, is assessed. While current AlphaFold2 models stored in AlphaFold DB still lack atomic resolution, they certainly provide useful information for a number of semiquantitative applications. However, in agreement with Shao and colleagues, it is reasonable to conclude that “experimentally determined crystal structures are more reliable than AlphaFold2-computed structure models and should be used preferentially whenever possible” (Shao et al., 2022).

## Data Availability

The original contributions presented in the study are included in the article/Supplementary Material, further inquiries can be directed to the corresponding authors.
