# Supplementary material for: Automated identification of chalcogen bonds in AlphaFold protein structure database files: is it possible?
Source: Front Mol Biosci. 2023 Jul 6;10:1155629. doi: 10.3389/fmolb.2023.1155629 (PMC10359982; doi:10.3389/fmolb.2023.1155629)
Supplement: Supplementary file 1 [file DataSheet1.PDF]

Automated identification of chalcogen bonds in AlphaFold protein structure database files: is it possible?

Oliviero Carugo[a] and Kristina Djinović-Carugo[b]

[a] Department of Chemistry, University of Pavia, Pavia, Italy & Department of Structural and Computational Biology, Max Perutz Labs, University of Vienna, Campus Vienna Biocenter 5, A-1030 Vienna, Austria (ORCID: 0000-0002-2924-9016)

[a] Department of Chemistry, University of Pavia, Pavia, Italy & Department of Structural and Computational Biology, Max Perutz Labs, University of Vienna, Campus Vienna Biocenter 5, A-1030 Vienna, Austria (ORCID: 0000-0002-2924-9016)

[b] European Molecular Biology Laboratory (EMBL) Grenoble, 71 Avenue des Martyrs, 38000 Grenoble, France & Department of Structural and Computational Biology, Max Perutz Labs, University of Vienna, Campus Vienna Biocenter 5, A-1030 Vienna, Austria & Department of Biochemistry, Faculty of Chemistry and Chemical Technology, University of Ljubljana, Večna pot 113, SI-1000 Ljubljana, Slovenia (ORCID: 0000-0003-0252-2972)

Supplementary materials

Table S1. Comparison between the experimental and computational stereochemistry of chalcogen bonds. Each chalcogen bond is described by two lines; the first reports the experimental data and the second the predicted data. In each line contains the following information: the accession code (in the Protein Data Bank and in AlphaFold DB); the atom covalently bound to the chalcogen atom; the chalcogen atom involved in the chalcogen bond; the nucleophilic atom involved in the chalcogen bond; and the variables *d* and α, described in Figure 1a, that characterize the chalcogen bond.

**Identification code**	**atom bound to chalcogen**	**chalcogen**	**nucleophile**	**d**	**α**
1bte	SG CYS A 41	SG CYS A 11	SG CYS A 59	3.66	12.1
AF-P27038-F1-model_v3.pdb	SG CYS A 60	SG CYS A 30	SG CYS A 78	4.20	9.3
1bte	CB CYS A 31	SG CYS A 31	SG CYS A 11	3.40	12.4
AF-P27038-F1-model_v3.pdb	CB CYS A 50	SG CYS A 50	SG CYS A 30	3.22	28.6
1bte	CB CYS A 59	SG CYS A 59	O GLU A 29	3.38	18.3
AF-P27038-F1-model_v3.pdb	CB CYS A 78	SG CYS A 78	O GLU A 48	3.97	17.6
1bte	SG CYS A 86	SG CYS A 91	O GLY A 88	3.05	6.9
AF-P27038-F1-model_v3.pdb	SG CYS A 105	SG CYS A 110	O GLY A 107	3.26	5.0
1eb6	SG CYS A 103	SG CYS A 85	OD2 ASP A 80	3.22	4.9
AF-P46076-F1-model_v3.pdb	SG CYS A 278	SG CYS A 260	OD2 ASP A 255	3.64	24.7

1ezg	SG CYS A 19	SG CYS A 9	O GLN A 2	3.31	10.1
AF-O16119-F1-model_v3.pdb	SG CYS A 46	SG CYS A 36	O GLN A 29	3.13	6.8
1f94	SG CYS A 24	SG CYS A 3	SG CYS A 39	3.46	10.3
AF-P81782-F1-model_v3.pdb	SG CYS A 24	SG CYS A 3	SG CYS A 39	3.74	12.9
1f94	CB CYS A 17	SG CYS A 17	SG CYS A 3	3.23	13.8
AF-P81782-F1-model_v3.pdb	CB CYS A 17	SG CYS A 17	SG CYS A 3	3.23	16.6
1f94	SG CYS A 56	SG CYS A 61	O THR A 58	2.90	1.0
AF-P81782-F1-model_v3.pdb	SG CYS A 56	SG CYS A 61	O THR A 58	2.98	12.6
1g1t	SG CYS A 90	SG CYS A 109	O ASP A 87	3.30	17.0
AF-P16581-F1-model_v3.pdb	SG CYS A 111	SG CYS A 130	O ASP A 108	5.13	43.0
1g1t	CB CYS A 144	SG CYS A 144	O GLY A 150	3.37	22.5
AF-P16581-F1-model_v3.pdb	CB CYS A 165	SG CYS A 165	O GLY A 171	3.59	27.8
1gu2	SG CYS A 104	SG CYS A 96	O PHE A 92	3.35	7.2
AF-Q9RQB9-F1-model_v3.pdb	SG CYS A 124	SG CYS A 116	O PHE A 112	3.23	5.0
1ijy	SG CYS A 16	SG CYS A 62	O LYS A 58	3.23	21.3
AF-Q61091-F1-model_v3.pdb	SG CYS A 43	SG CYS A 89	O LYS A 85	3.31	17.1
1ijy	CB CYS A 84	SG CYS A 84	O MET A 106	3.16	24.0
AF-Q61091-F1-model_v3.pdb	CB CYS A 111	SG CYS A 111	O MET A 133	3.41	26.5
1ikp	SG CYS A 287	SG CYS A 265	O ALA A 261	3.32	21.6
AF-P11439-F1-model_v3.pdb	SG CYS A 312	SG CYS A 290	O ALA A 286	3.45	22.5
1itx	CB CYS A 101	SG CYS A 101	O ILE A 85	3.28	20.2
AF-P20533-F1-model_v3.pdb	CB CYS A 101	SG CYS A 101	O ILE A 85	3.39	18.9
1l9l	SG CYS A 7	SG CYS A 70	O ALA A 66	3.29	22.9
AF-P22749-F1-model_v3.pdb	SG CYS A 69	SG CYS A 132	O ALA A 128	3.03	38.8
1llf	SG CYS A 97	SG CYS A 60	O TYR A 156	3.33	24.8
AF-P32947-F1-model_v3.pdb	SG CYS A 112	SG CYS A 75	O TYR A 171	3.23	31.3
1ojj	CB CYS A 63	SG CYS A 63	SG CYS A 73	3.43	10.1
AF-P56680-F1-model_v3.pdb	CB CYS A 63	SG CYS A 63	SG CYS A 73	3.56	10.4
1ojj	CB CYS A 194	SG CYS A 194	SG CYS A 223	3.23	20.8
AF-P56680-F1-model_v3.pdb	CB CYS A 194	SG CYS A 194	SG CYS A 223	3.30	34.6
1p3c	SG CYS A 48	SG CYS A 32	O SER A 31	3.22	8.1
AF-Q9EXR9-F1-model_v3.pdb	SG CYS A 136	SG CYS A 120	O SER A 119	3.43	4.4
1pwa	SG CYS A 102	SG CYS A 120	O SER A 116	3.09	3.7
AF-O95750-F1-model_v3.pdb	SG CYS A 102	SG CYS A 120	O SER A 116	3.32	10.2
1qft	SG CYS A 48	SG CYS A 169	O THR A 166	3.26	12.9

AF-O77421-F1-model_v3.pdb	SG CYS A 67	SG CYS A 188	O THR A 185	3.46	15.0
1qqf	SG CYS A1158	SG CYS A1101	OE1 GLN A1161	3.35	17.1
AF-P01026-F1-model_v3.pdb	SG CYS A1158	SG CYS A1101	OE1 GLN A1161	3.93	18.4
1ryo	SG CYS A 179	SG CYS A 161	O PRO A 160	3.14	18.9
AF-P02787-F1-model_v3.pdb	SG CYS A 198	SG CYS A 180	O PRO A 179	3.41	23.7
1ryo	SG CYS A 161	SG CYS A 179	OG SER A 44	3.37	21.8
AF-P02787-F1-model_v3.pdb	SG CYS A 180	SG CYS A 198	OG SER A 63	5.90	34.0
1ryo	CB CYS A 177	SG CYS A 177	O LEU A 170	3.18	22.9
AF-P02787-F1-model_v3.pdb	CB CYS A 196	SG CYS A 196	O LEU A 189	3.49	12.7
1vf8	SG CYS A 28	SG CYS A 373	OG SER A 370	3.16	11.4
AF-O35744-F1-model_v3.pdb	SG CYS A 49	SG CYS A 394	OG SER A 391	5.22	85.9
1ypq	CB CYS A 155	SG CYS A 155	O PRO A 143	3.37	16.0
AF-P78380-F1-model_v3.pdb	CB CYS A 155	SG CYS A 155	O PRO A 143	3.62	16.7
1ypq	SG CYS A 243	SG CYS A 256	O GLY A 241	3.20	23.1
AF-P78380-F1-model_v3.pdb	SG CYS A 243	SG CYS A 256	O GLY A 241	3.33	29.0
2a8f	SG CYS A 95	SG CYS A 51	O TYR A 47	2.97	22.9
AF-P15569-F1-model_v3.pdb	SG CYS A 95	SG CYS A 51	O TYR A 47	3.00	38.2
2ahn	CB CYS A 221	SG CYS A 221	OH TYR A 11	3.34	6.3
AF-P50694-F1-model_v3.pdb	CB CYS A 244	SG CYS A 244	OH TYR A 34	3.61	13.6
2ahn	CB CYS A 127	SG CYS A 127	O THR A 124	3.23	17.7
AF-P50694-F1-model_v3.pdb	CB CYS A 150	SG CYS A 150	O THR A 147	3.38	25.7
2ahn	SG CYS A 132	SG CYS A 193	O ALA A 192	3.38	13.1
AF-P50694-F1-model_v3.pdb	SG CYS A 155	SG CYS A 216	O ALA A 215	3.33	8.9
2cdc	SG CYS A 181	SG CYS A 174	O THR A 178	3.24	6.8
AF-O93715-F1-model_v3.pdb	SG CYS A 181	SG CYS A 174	O THR A 178	3.42	18.9
2czq	SG CYS A 78	SG CYS A 6	O LEU A 201	3.37	6.2
AF-Q874E9-F1-model_v3.pdb	SG CYS A 112	SG CYS A 40	O LEU A 235	3.76	11.8
2hkm	SG CYS A 242	SG CYS A 225	O ALA A 222	3.39	16.1
AF-P84888-F1-model_v3.pdb	SG CYS A 199	SG CYS A 182	O ALA A 179	3.71	14.8
2hox	SG CYS A 20	SG CYS A 39	O PRO A 48	3.33	24.8
AF-Q01594-F1-model_v3.pdb	SG CYS A 58	SG CYS A 77	O PRO A 86	3.35	12.0
2hox	CB CYS A 44	SG CYS A 44	O SER A 58	3.17	17.1
AF-Q01594-F1-model_v3.pdb	CB CYS A 82	SG CYS A 82	O SER A 96	3.17	17.2

2j8b	SG CYS A 26	SG CYS A 3	SG CYS A 19	3.28	12.8
AF-P13987-F1-model_v3.pdb	SG CYS A 51	SG CYS A 28	SG CYS A 44	3.36	11.7
2j8b	SG CYS A 64	SG CYS A 69	O LYS A 66	2.87	3.2
AF-P13987-F1-model_v3.pdb	SG CYS A 89	SG CYS A 94	O LYS A 91	2.94	11.1
2or7	CB CYS A 38	SG CYS A 38	O VAL A 102	3.31	24.9
AF-Q8R183-F1-model_v3.pdb	CB CYS A 56	SG CYS A 56	O VAL A 120	3.67	35.1
2q9o	SG CYS A 540	SG CYS A 114	O TRP A 384	3.14	19.4
AF-Q70KY3-F1-model_v3.pdb	SG CYS A 590	SG CYS A 164	O TRP A 434	3.24	15.1
2qes	CB CYS A 258	SG CYS A 258	O LYS A 33	3.16	14.7
AF-P84854-F1-model_v3.pdb	CB CYS A 258	SG CYS A 258	O LYS A 33	3.46	23.4
2rdk	SG CYS A 22	SG CYS A 8	O PHE A 4	3.16	9.4
AF-P81180-F1-model_v3.pdb	SG CYS A 22	SG CYS A 8	O PHE A 4	3.29	14.8
2rdk	SG CYS A 73	SG CYS A 58	O PHE A 54	3.16	11.1
AF-P81180-F1-model_v3.pdb	SG CYS A 73	SG CYS A 58	O PHE A 54	3.35	11.0
2uvo	CB CYS A 17	SG CYS A 17	O ALA A 39	3.28	18.8
AF-P10968-F1-model_v3.pdb	CB CYS A 43	SG CYS A 43	O ALA A 65	3.30	30.9
2uvo	SG CYS A 40	SG CYS A 35	O TYR A 30	3.33	8.7
AF-P10968-F1-model_v3.pdb	SG CYS A 66	SG CYS A 61	O TYR A 56	3.44	8.7
2uvo	CB CYS A 55	SG CYS A 55	SG CYS A 46	3.47	22.2
AF-P10968-F1-model_v3.pdb	CB CYS A 81	SG CYS A 81	SG CYS A 72	3.50	20.2
2uvo	CB CYS A 78	SG CYS A 78	O GLY A 80	3.12	20.9
AF-P10968-F1-model_v3.pdb	CB CYS A 104	SG CYS A 104	O GLY A 106	3.29	29.1
2uvo	CB CYS A 98	SG CYS A 98	SG CYS A 89	3.48	18.9
AF-P10968-F1-model_v3.pdb	CB CYS A 124	SG CYS A 124	SG CYS A 115	3.49	16.3
2uvo	CB CYS A 103	SG CYS A 103	O ALA A 125	3.26	17.0
AF-P10968-F1-model_v3.pdb	CB CYS A 129	SG CYS A 129	O ALA A 151	3.21	29.7
2uvo	SG CYS A 126	SG CYS A 121	O PHE A 116	3.37	10.6
AF-P10968-F1-model_v3.pdb	SG CYS A 152	SG CYS A 147	O PHE A 142	3.40	8.3
2uvo	CB CYS A 141	SG CYS A 141	SG CYS A 132	3.62	19.6
AF-P10968-F1-model_v3.pdb	CB CYS A 167	SG CYS A 167	SG CYS A 158	3.62	18.7
2uvo	SG CYS A 169	SG CYS A 164	O TYR A 159	3.35	11.8
AF-P10968-F1-model_v3.pdb	SG CYS A 195	SG CYS A 190	O TYR A 185	3.51	12.8
2x5x	SG CYS A 246	SG CYS A 255	O TRP A 252	3.18	11.9
AF-Q939Q9-F1-model_v3.pdb	SG CYS A 284	SG CYS A 293	O TRP A 290	3.23	9.3
3arq	SG CYS A 196	SG CYS A 217	O LEU A 213	3.32	17.7

AF-Q9AMP1-F1-model_v3.pdb	SG CYS A 196	SG CYS A 217	O LEU A 213	3.38	26.9
3b4n	SG CYS A 143	SG CYS A 193	O ALA A 172	3.25	13.1
AF-O50325-F1-model_v3.pdb	SG CYS A 143	SG CYS A 193	O ALA A 172	4.28	51.0
3bqp	SG CYS A 8	SG CYS A 72	O PRO A 68	3.24	16.5
AF-P07602-F1-model_v3.pdb	SG CYS A 412	SG CYS A 476	O PRO A 472	3.00	17.6
3c1u	SG CYS A 232	SG CYS A 214	O LYS A 210	3.22	16.4
AF-Q00017-F1-model_v3.pdb	SG CYS A 249	SG CYS A 231	O LYS A 227	3.31	13.7
3ca7	SG CYS A 63	SG CYS A 82	O GLN A 91	3.37	8.0
AF-Q01083-F1-model_v3.pdb	SG CYS A 91	SG CYS A 110	O GLN A 119	3.74	2.9
3dfj	SG CYS A 86	SG CYS A 70	O VAL A 69	3.10	9.8
AF-Q16651-F1-model_v3.pdb	SG CYS A 86	SG CYS A 70	O VAL A 69	3.23	6.5
3dfj	SG CYS A 168	SG CYS A 244	OG1 THR A 253	2.92	9.2
AF-Q16651-F1-model_v3.pdb	SG CYS A 168	SG CYS A 244	OG1 THR A 253	3.09	13.2
3ef3	CB CYS A 31	SG CYS A 31	O LYS A 108	3.20	20.1
AF-P00590-F1-model_v3.pdb	CB CYS A 47	SG CYS A 47	O LYS A 124	3.09	16.9
3f5v	SG CYS A 103	SG CYS A 65	O GLU A 61	3.37	20.4
AF-Q3HWZ5-F1-model_v3.pdb	SG CYS A 183	SG CYS A 145	O GLU A 141	3.60	21.8
3ftd	CB CYS A 90	SG CYS A 90	O ASN A 117	3.36	12.6
AF-O67680-F1-model_v3.pdb	CB CYS A 90	SG CYS A 90	O ASN A 117	3.59	16.6
3g7n	SG CYS A 228	SG CYS A 220	O GLU A 207	3.30	6.5
AF-Q9HFW6-F1-model_v3.pdb	SG CYS A 228	SG CYS A 220	O GLU A 207	3.38	12.8
3h31	CB CYS A 50	SG CYS A 50	O THR A 4	3.21	5.7
AF-C4P582-F1-model_v3.pdb	CB CYS A 103	SG CYS A 103	O THR A 57	3.40	10.2
3k1w	CB CYS A 217	SG CYS A 217	O MET A 205	3.34	14.8
AF-P00797-F1-model_v3.pdb	CB CYS A 283	SG CYS A 283	O MET A 271	3.30	24.0
3k1w	SG CYS A 259	SG CYS A 296	O PHE A 286	3.39	22.8
AF-P00797-F1-model_v3.pdb	SG CYS A 325	SG CYS A 362	O PHE A 352	3.35	22.4
3mzq	SG CYS A 84	SG CYS A 26	OG1 THR A 99	3.23	6.7
AF-P61823-F1-model_v3.pdb	SG CYS A 110	SG CYS A 52	OG1 THR A 125	3.45	9.7
3neq	SG CYS A 59	SG CYS A 64	O THR A 61	2.93	1.8
AF-Q8QGR0-F1-model_v3.pdb	SG CYS A 79	SG CYS A 84	O THR A 81	3.04	4.2
3nhi	SG CYS A 193	SG CYS A 160	O PHE A 156	3.06	23.9

AF-Q95NY5-F1-model_v3.pdb	SG CYS A 211	SG CYS A 178	O PHE A 174	3.08	22.1
3nhi	CB CYS A 234	SG CYS A 234	O ASP A 241	3.08	18.7
AF-Q95NY5-F1-model_v3.pdb	CB CYS A 252	SG CYS A 252	O ASP A 259	3.30	18.9
3nrf	CB CYS A 125	SG CYS A 125	O SER A 122	3.33	8.2
AF-Q9I2H0-F1-model_v3.pdb	CB CYS A 125	SG CYS A 125	O SER A 122	3.51	23.7
3om0	SG CYS A 17	SG CYS A 273	O ASN A 272	3.23	15.2
AF-Q63273-F1-model_v3.pdb	SG CYS A 36	SG CYS A 292	O ASN A 291	3.82	115.3
3qsd	SG CYS A 141	SG CYS A 97	OG SER A 99	3.34	19.3
AF-Q8MNY2-F1-model_v3.pdb	SG CYS A 158	SG CYS A 114	OG SER A 116	3.30	27.9
3qsd	SG CYS A 170	SG CYS A 203	O THR A 209	3.13	21.6
AF-Q8MNY2-F1-model_v3.pdb	SG CYS A 187	SG CYS A 220	O THR A 226	3.37	40.0
3soj	CB CYS A 132	SG CYS A 132	O SER A 115	3.13	22.2
AF-Q5NGF6-F1-model_v3.pdb	CB CYS A 140	SG CYS A 140	O SER A 123	5.37	117.3
3suj	SG CYS A 78	SG CYS A 41	O ILE A 101	3.24	12.7
AF-B2C3H7-F1-model_v3.pdb	SG CYS A 78	SG CYS A 41	O ILE A 101	3.31	17.6
3suj	SG CYS A 41	SG CYS A 78	O SER A 75	3.34	20.6
AF-B2C3H7-F1-model_v3.pdb	SG CYS A 41	SG CYS A 78	O SER A 75	3.42	23.4
3uci	SG CYS A 4	SG CYS A 19	O THR A 18	2.87	7.8
AF-P30403-F1-model_v3.pdb	SG CYS A 411	SG CYS A 426	O THR A 425	4.19	106.1
3uci	CB CYS A 27	SG CYS A 27	O LYS A 37	3.39	15.2
AF-P30403-F1-model_v3.pdb	CB CYS A 434	SG CYS A 434	O LYS A 444	3.31	30.0
3uci	CB CYS A 32	SG CYS A 32	SG CYS A 45	3.47	7.1
AF-P30403-F1-model_v3.pdb	CB CYS A 439	SG CYS A 439	SG CYS A 452	3.53	12.0
3v38	SG CYS A 156	SG CYS A 155	O LYS A 152	3.22	8.2
AF-P29068-F1-model_v3.pdb	SG CYS A 254	SG CYS A 253	O LYS A 250	3.09	9.5
3vtg	SG CYS A 199	SG CYS A 50	O GLY A 18	3.35	24.4
AF-P31580-F1-model_v3.pdb	SG CYS A 269	SG CYS A 120	O GLY A 88	3.84	33.5
3vup	SG CYS A 244	SG CYS A 177	OE2 GLU A 175	3.21	22.8
AF-E5RSM0-F1-model_v3.pdb	SG CYS A 262	SG CYS A 195	OE2 GLU A 193	5.18	5.5
3w7t	SG CYS A 594	SG CYS A 617	OD1 ASN A 615	3.14	22.2
AF-P42592-F1-model_v3.pdb	SG CYS A 617	SG CYS A 640	OD1 ASN A 638	3.33	28.6
3wli	SG CYS A 518	SG CYS A 513	O VAL A 509	3.11	10.5

AF-Q9XEI3-F1-model_v3.pdb	SG CYS A 543	SG CYS A 538	O VAL A 534	3.09	12.5
3wmt	CB CYS A 76	SG CYS A 76	O GLY A 128	3.15	18.7
AF-Q2UP89-F1-model_v3.pdb	CB CYS A 76	SG CYS A 76	O GLY A 128	3.52	32.9
3x2g	SG CYS A 54	SG CYS A 26	O THR A 84	3.07	16.2
AF-B3Y002-F1-model_v3.pdb	SG CYS A 80	SG CYS A 52	O THR A 110	3.07	16.5
3x2g	SG CYS A 26	SG CYS A 54	O GLY A 51	3.32	16.7
AF-B3Y002-F1-model_v3.pdb	SG CYS A 52	SG CYS A 80	O GLY A 77	3.40	18.5
3zxc	CB CYS A 50	SG CYS A 50	SG CYS A 40	3.57	16.2
AF-G4V4F9-F1-model_v3.pdb	CB CYS A 69	SG CYS A 69	SG CYS A 59	3.52	16.7
3zzo	SG CYS A 87	SG CYS A 27	SG CYS A 83	3.36	15.1
AF-Q9VAK8-F1-model_v3.pdb	SG CYS A 87	SG CYS A 27	SG CYS A 83	3.40	13.9
4bct	SG CYS A 146	SG CYS A 196	O ARG A 195	3.34	10.9
AF-P81370-F1-model_v3.pdb	SG CYS A 146	SG CYS A 196	O ARG A 195	3.34	11.3
4br9	SG CYS A 39	SG CYS A 44	O SER A 43	3.27	15.1
AF-Q5ZUA2-F1-model_v3.pdb	SG CYS A 39	SG CYS A 44	O SER A 43	3.44	20.3
4bww	CB CYS A 26	SG CYS A 26	O GLU A 2	3.24	20.0
AF-P00282-F1-model_v3.pdb	CB CYS A 46	SG CYS A 46	O GLU A 22	4.37	34.6
4dm5	CB CYS A 85	SG CYS A 85	O ASN A 54	3.29	24.6
AF-Q9HUT7-F1-model_v3.pdb	CB CYS A 102	SG CYS A 102	O ASN A 71	3.44	22.3
4dm5	SG CYS A 53	SG CYS A 85	OG SER A 55	3.13	13.2
AF-Q9HUT7-F1-model_v3.pdb	SG CYS A 70	SG CYS A 102	OG SER A 72	3.14	22.3
4f0j	CB CYS A 105	SG CYS A 105	O PHE A 77	3.17	19.9
AF-Q9HZF5-F1-model_v3.pdb	CB CYS A 105	SG CYS A 105	O PHE A 77	3.29	19.5
4ftf	SG CYS A 111	SG CYS A 74	O VAL A 70	3.26	18.0
AF-Q9KRD9-F1-model_v3.pdb	SG CYS A 160	SG CYS A 123	O VAL A 119	3.26	24.7
4hwv	SG CYS A 122	SG CYS A 164	O ASP A 160	3.36	16.4
AF-A1TSQ3-F1-model_v3.pdb	SG CYS A 122	SG CYS A 164	O ASP A 160	3.30	15.5
4hwv	SG CYS A 425	SG CYS A 434	O SER A 430	3.03	8.8
AF-A1TSQ3-F1-model_v3.pdb	SG CYS A 425	SG CYS A 434	O SER A 430	3.59	17.9
4io2	SG CYS A 193	SG CYS A 247	O ASN A 189	3.27	24.8
AF-E9P5T5-F1-model_v3.pdb	SG CYS A 757	SG CYS A 811	O ASN A 753	3.33	20.0
4ipu	CB CYS A 158	SG CYS A 158	O LYS A 131	3.35	17.5
AF-Q59652-F1-model_v3.pdb	CB CYS A 167	SG CYS A 167	O LYS A 140	3.42	22.5
4k3l	CB CYS A 260	SG CYS A 260	O ASN A 335	3.35	17.1
AF-P0A988-F1-model_v3.pdb	CB CYS A 260	SG CYS A 260	O ASN A 335	6.10	123.7

4n30	SG CYS A 157	SG CYS A 111	O ARG A 107	3.23	12.5
AF-Q7WY37-F1-model_v3.pdb	SG CYS A 157	SG CYS A 111	O ARG A 107	3.25	16.4
4n9o	SG CYS A 179	SG CYS A 214	O VAL A 210	3.15	20.3
AF-P04156-F1-model_v3.pdb	SG CYS A 179	SG CYS A 214	O VAL A 210	3.22	20.1
4nds	SG CYS A 57	SG CYS A 27	O ALA A 23	3.19	22.7
AF-A7UNK4-F1-model_v3.pdb	SG CYS A 57	SG CYS A 27	O ALA A 23	3.28	25.2
4nsv	SG CYS A 80	SG CYS A 12	O ASP A 9	3.26	23.4
AF-Q7M135-F1-model_v3.pdb	SG CYS A 80	SG CYS A 12	O ASP A 9	3.33	26.2
4nsv	SG CYS A 58	SG CYS A 36	O TRP A 35	3.00	9.1
AF-Q7M135-F1-model_v3.pdb	SG CYS A 58	SG CYS A 36	O TRP A 35	3.35	3.4
4oel	SG CYS A 307	SG CYS A 267	O GLU A 263	3.32	11.6
AF-P53634-F1-model_v3.pdb	SG CYS A 331	SG CYS A 291	O GLU A 287	3.40	16.8
4opb	CB CYS A 185	SG CYS A 185	O TYR A 193	3.32	20.3
AF-Q2U8Y3-F1-model_v3.pdb	CB CYS A 231	SG CYS A 231	O TYR A 239	3.24	22.4
4rax	SG CYS A2441	SG CYS A2437	O LEU A2434	3.21	3.4
AF-E2JF22-F1-model_v3.pdb	SG CYS A2441	SG CYS A2437	O LEU A2434	3.38	8.2
4ruq	SG CYS A 100	SG CYS A 153	OD1 ASN A 102	3.39	17.3
AF-P68512-F1-model_v3.pdb	SG CYS A 100	SG CYS A 153	OD1 ASN A 102	3.29	18.5
4uzq	CB CYS A 449	SG CYS A 449	O ALA A 385	3.30	23.0
AF-Q6P988-F1-model_v3.pdb	CB CYS A 449	SG CYS A 449	O ALA A 385	3.59	27.4
4uzq	SG CYS A 440	SG CYS A 445	O TRP A 442	2.90	9.6
AF-Q6P988-F1-model_v3.pdb	SG CYS A 440	SG CYS A 445	O TRP A 442	2.87	27.2
4wk7	SG CYS A 407	SG CYS A 377	O HIS A 371	3.17	13.0
AF-O75173-F1-model_v3.pdb	SG CYS A 407	SG CYS A 377	O HIS A 371	3.42	20.6
4xmh	CB CYS A 124	SG CYS A 124	OD1 ASP A 131	3.40	15.4
AF-Q6PQK2-F1-model_v3.pdb	CB CYS A 144	SG CYS A 144	OD1 ASP A 151	3.38	12.4
4xoj	CB CYS A 22	SG CYS A 22	O LYS A 151	3.38	16.2
AF-P00760-F1-model_v3.pdb	CB CYS A 30	SG CYS A 30	O LYS A 159	3.38	21.7
4xoj	SG CYS A 56	SG CYS A 40	O PHE A 39	3.02	5.5
AF-P00760-F1-model_v3.pdb	SG CYS A 64	SG CYS A 48	O PHE A 47	3.12	8.3

4xoj	CB CYS A 212	SG CYS A 212	OE1 GLN A 189	3.18	17.3
AF-P00760-F1-model_v3.pdb	CB CYS A 220	SG CYS A 220	OE1 GLN A 197	3.25	30.5
5ajo	SG CYS A 354	SG CYS A 126	O HIS A 123	3.37	24.7
AF-Q10471-F1-model_v3.pdb	SG CYS A 354	SG CYS A 126	O HIS A 123	3.52	24.1
5ajo	SG CYS A 423	SG CYS A 345	O ARG A 341	3.15	12.8
AF-Q10471-F1-model_v3.pdb	SG CYS A 423	SG CYS A 345	O ARG A 341	3.12	21.1
5ajo	CB CYS A 473	SG CYS A 473	O ASN A 455	3.17	23.1
AF-Q10471-F1-model_v3.pdb	CB CYS A 473	SG CYS A 473	O ASN A 455	3.32	29.0
5dj7	SG CYS A 43	SG CYS A 27	O PHE A 26	3.17	10.1
AF-Q54137-F1-model_v3.pdb	SG CYS A 85	SG CYS A 69	O PHE A 68	3.27	12.4
5dj7	CB CYS A 199	SG CYS A 199	OE1 GLN A 176	3.20	10.0
AF-Q54137-F1-model_v3.pdb	CB CYS A 241	SG CYS A 241	OE1 GLN A 218	3.39	25.3
5dj7	SG CYS A 175	SG CYS A 199	O GLY A 198	3.38	6.8
AF-Q54137-F1-model_v3.pdb	SG CYS A 217	SG CYS A 241	O GLY A 240	3.42	7.8
5ftz	SG CYS A 51	SG CYS A 43	O ARG A 39	3.31	13.3
AF-D6EWM4-F1-model_v3.pdb	SG CYS A 51	SG CYS A 43	O ARG A 39	3.25	16.4
5glx	SG CYS A 136	SG CYS A 13	OE1 GLN A 139	3.32	23.2
AF-G2QVH7-F1-model_v3.pdb	SG CYS A 157	SG CYS A 34	OE1 GLN A 160	3.40	32.8
5glx	SG CYS A 87	SG CYS A 18	O THR A 110	3.28	16.2
AF-G2QVH7-F1-model_v3.pdb	SG CYS A 108	SG CYS A 39	O THR A 131	3.34	22.0
5glx	SG CYS A 88	SG CYS A 200	O GLN A 28	3.02	18.2
AF-G2QVH7-F1-model_v3.pdb	SG CYS A 109	SG CYS A 221	O GLN A 49	3.05	25.6
5hha	SG CYS A 222	SG CYS A 242	O LYS A 241	3.39	21.6
AF-Q9I185-F1-model_v3.pdb	SG CYS A 222	SG CYS A 242	O LYS A 241	3.59	24.0
5kzz	SG CYS A 100	SG CYS A 51	SD MET A 104	3.64	18.8
AF-Q98SW5-F1-model_v3.pdb	SG CYS A 100	SG CYS A 51	SD MET A 104	3.88	22.3
5m5z	CB CYS A 569	SG CYS A 569	O GLN A 554	3.25	7.3
AF-D8UU87-F1-model_v3.pdb	CB CYS A 569	SG CYS A 569	O GLN A 554	3.68	9.6
5mfa	SG CYS A 398	SG CYS A 387	O PHE A 532	3.07	19.9
AF-P05164-F1-model_v3.pdb	SG CYS A 398	SG CYS A 387	O PHE A 532	3.39	24.1

5n7q	CB CYS A 49	SG CYS A 49	OG1 THR A 51	3.30	21.2
AF-V5HCK7-F1-model_v3.pdb	CB CYS A 92	SG CYS A 92	OG1 THR A 94	3.30	18.0
5ns9	CB CYS A 206	SG CYS A 206	O GLU A 260	3.37	19.1
AF-P19491-F1-model_v3.pdb	CB CYS A 739	SG CYS A 739	O GLU A 793	4.84	19.1
5o9q	SG CYS A 89	SG CYS A 118	O LEU A 83	3.29	20.0
AF-Q06135-F1-model_v3.pdb	SG CYS A 89	SG CYS A 118	O LEU A 83	3.26	22.6
5o9q	SG CYS A 367	SG CYS A 231	O TYR A 228	3.17	21.9
AF-Q06135-F1-model_v3.pdb	SG CYS A 367	SG CYS A 231	O TYR A 228	3.19	29.2
5opf	SG CYS A 50	SG CYS A 70	OD2 ASP A 67	3.08	11.5
AF-D9SZQ3-F1-model_v3.pdb	SG CYS A 50	SG CYS A 70	OD2 ASP A 67	3.05	10.0
5ugd	SG CYS A 604	SG CYS A 588	O PHE A 587	3.04	3.4
AF-P00747-F1-model_v3.pdb	SG CYS A 623	SG CYS A 607	O PHE A 606	3.36	5.9
5xbu	SG CYS A 69	SG CYS A 175	O CYS A 33	3.12	14.6
AF-A7KMF0-F1-model_v3.pdb	SG CYS A 85	SG CYS A 191	O CYS A 49	3.21	12.6
5xkx	SG CYS A 394	SG CYS A 374	O TYR A 262	3.35	21.5
AF-N8X9V6-F1-model_v3.pdb	SG CYS A 394	SG CYS A 374	O TYR A 262	3.30	24.0
5yre	SG CYS A 150	SG CYS A 78	O ASN A 75	3.10	15.2
AF-B6F0T7-F1-model_v3.pdb	SG CYS A 150	SG CYS A 78	O ASN A 75	3.22	13.7
6a5d	SG CYS A 65	SG CYS A 75	O PRO A 71	3.06	3.3
AF-Q9FKT1-F1-model_v3.pdb	SG CYS A 65	SG CYS A 75	O PRO A 71	3.49	14.8
6a5d	SG CYS A 129	SG CYS A 84	O LYS A 80	3.23	20.0
AF-Q9FKT1-F1-model_v3.pdb	SG CYS A 129	SG CYS A 84	O LYS A 80	3.30	33.8
6ac5	SG CYS A 601	SG CYS A 656	O ALA A 652	3.26	23.1
AF-Q13546-F1-model_v3.pdb	SG CYS A 601	SG CYS A 656	O ALA A 652	3.16	105.0
6c74	SG CYS A 36	SG CYS A 44	O GLN A 41	3.23	17.1
AF-Q6SJQ7-F1-model_v3.pdb	SG CYS A 55	SG CYS A 63	O GLN A 60	3.25	18.7
6ej2	CB CYS A 564	SG CYS A 564	OE1 GLN A 562	3.37	6.1
AF-P56817-F1-model_v3.pdb	CB CYS A 216	SG CYS A 216	OE1 GLN A 214	3.59	13.3
6elc	SG CYS A 212	SG CYS A 230	O ALA A 226	3.34	18.8
AF-B3GVK1-F1-model_v3.pdb	SG CYS A 212	SG CYS A 230	O ALA A 226	3.35	35.0
6gbi	SG CYS A 77	SG CYS A 49	SG CYS A 96	3.56	4.2
AF-Q86Y78-F1-model_v3.pdb	SG CYS A 77	SG CYS A 49	SG CYS A 96	3.81	5.7

6gbi	CB CYS A 70	SG CYS A 70	SG CYS A 49	3.51	13.7
AF-Q86Y78-F1-model_v3.pdb	CB CYS A 70	SG CYS A 70	SG CYS A 49	3.42	24.7
6gbi	SG CYS A 102	SG CYS A 121	O SER A 120	3.38	5.7
AF-Q86Y78-F1-model_v3.pdb	SG CYS A 102	SG CYS A 121	O SER A 120	3.60	10.9
6gbi	SG CYS A 122	SG CYS A 127	O GLY A 124	3.16	7.1
AF-Q86Y78-F1-model_v3.pdb	SG CYS A 122	SG CYS A 127	O GLY A 124	3.46	15.3
6git	CB CYS A 217	SG CYS A 217	OG SER A 219	3.16	21.9
AF-C4PKL0-F1-model_v3.pdb	CB CYS A 237	SG CYS A 237	OG SER A 239	3.28	29.1
6h20	SG CYS A 66	SG CYS A 167	O SER A 63	2.98	18.1
AF-P96257-F1-model_v3.pdb	SG CYS A 66	SG CYS A 167	O SER A 63	5.46	86.5
6h5w	SG CYS A 550	SG CYS A 538	O HIS A 534	2.99	11.4
AF-P12821-F1-model_v3.pdb	SG CYS A1155	SG CYS A1143	O HIS A1139	3.04	18.7
6i0i	SG CYS A 31	SG CYS A 46	O ALA A 42	3.31	20.2
AF-P83544-F1-model_v3.pdb	SG CYS A 69	SG CYS A 84	O ALA A 80	3.23	24.0
6i44	SG CYS A 2	SG CYS A 85	O LEU A 82	3.36	14.9
AF-P03952-F1-model_v3.pdb	SG CYS A 21	SG CYS A 104	O LEU A 101	3.51	17.7
6i44	SG CYS A 416	SG CYS A 400	O LEU A 399	3.26	4.1
AF-P03952-F1-model_v3.pdb	SG CYS A 435	SG CYS A 419	O LEU A 418	3.48	7.2
6j33	SG CYS A 644	SG CYS A 643	O THR A 642	3.22	21.8
AF-W9BQ28-F1-model_v3.pdb	SG CYS A 663	SG CYS A 662	O THR A 661	3.65	40.7
6jd8	SG CYS A 194	SG CYS A 152	O ASN A 148	3.22	8.6
AF-P07711-F1-model_v3.pdb	SG CYS A 211	SG CYS A 169	O ASN A 165	3.27	8.4
6jd8	CB CYS A 252	SG CYS A 252	O ASP A 233	3.26	10.9
AF-P07711-F1-model_v3.pdb	CB CYS A 269	SG CYS A 269	O ASP A 250	3.24	9.9
6jk4	SG CYS A 69	SG CYS A 100	O LEU A 97	3.37	15.1
AF-P84493-F1-model_v3.pdb	SG CYS A 86	SG CYS A 117	O LEU A 114	4.03	49.2
6le8	SG CYS A 321	SG CYS A 385	OD2 ASP A 380	3.17	12.2
AF-Q11174-F1-model_v3.pdb	SG CYS A 341	SG CYS A 405	OD2 ASP A 400	3.47	25.2
6sxt	SG CYS A 86	SG CYS A 81	O GLN A 77	3.25	18.3
AF-Q8NK89-F1-model_v3.pdb	SG CYS A 86	SG CYS A 81	O GLN A 77	3.31	20.5
6uax	CB CYS A 221	SG CYS A 221	O GLY A 223	3.27	24.4
AF-A9GMG4-F1-model_v3.pdb	CB CYS A 221	SG CYS A 221	O GLY A 223	3.36	26.1
6ub5	SG CYS A 158	SG CYS A 154	O MET A 150	3.35	19.2

AF-G9M5R4-F1-model_v3.pdb	SG CYS A 158	SG CYS A 154	O MET A 150	3.35	22.6
6x0t	SG CYS A 413	SG CYS A 397	O PHE A 396	3.18	5.2
AF-P00748-F1-model_v3.pdb	SG CYS A 413	SG CYS A 397	O PHE A 396	3.46	0.7
6xwe	SG CYS A 152	SG CYS A 90	SG CYS A 154	3.66	20.8
AF-Q6UD73-F1-model_v3.pdb	SG CYS A 152	SG CYS A 90	SG CYS A 154	3.38	29.9
6ya1	CB CYS A 303	SG CYS A 303	O TYR A 330	3.06	19.8
AF-P21347-F1-model_v3.pdb	CB CYS A 510	SG CYS A 510	O TYR A 537	5.10	54.0
6zzb	SG CYS A 126	SG CYS A 213	OD1 ASP A 218	3.01	6.2
AF-Q70CD0-F1-model_v3.pdb	SG CYS A 126	SG CYS A 213	OD1 ASP A 218	3.12	21.3
7aah	SG CYS A 171	SG CYS A 53	OH TYR A 146	3.30	16.3
AF-Q8WU39-F1-model_v3.pdb	SG CYS A 171	SG CYS A 53	OH TYR A 146	3.34	16.3
7esw	CB CYS A 51	SG CYS A 51	OD1 ASN A 48	3.38	20.4
AF-G9MQD3-F1-model_v3.pdb	CB CYS A 51	SG CYS A 51	OD1 ASN A 48	3.49	17.3
7nx1	CB CYS A 179	SG CYS A 179	O ALA A 167	3.37	14.3
AF-P29376-F1-model_v3.pdb	CB CYS A 179	SG CYS A 179	O ALA A 167	4.21	24.0
7ofv	CB CYS A 108	SG CYS A 108	O LYS A 119	3.33	14.6
AF-P54764-F1-model_v3.pdb	CB CYS A 108	SG CYS A 108	O LYS A 119	3.65	17.4
